# Study protocol of the Berlin Research Initiative for Diagnostics, Genetics and Environmental Factors in Schizophrenia (BRIDGE-S)

**DOI:** 10.1186/s12888-022-04447-4

**Published:** 2023-01-12

**Authors:** Alice Braun, Julia Kraft, Stephan Ripke

**Affiliations:** 1 Department of Psychiatry and Psychotherapy, Charité – Universitätsmedizin Berlin, corporate member of Freie Universität Berlin and Humboldt Universität zu Berlin, Charitéplatz 1, 10117 Berlin, Germany; 2 Stanley Center for Psychiatric Research, Broad Institute of MIT and Harvard, Cambridge, MA USA

**Keywords:** GWAS, Gene-environment interaction, Schizophrenia, Psychosis, Psychiatric genetics, Risk-prediction

## Abstract

**Background:**

Large-scale collaborative efforts in the field of psychiatric genetics have made substantial progress in unraveling the biological architecture of schizophrenia (SCZ). Although both genetic and environmental factors are known to play a role in schizophrenia etiology our mechanistic understanding of how they shape risk, resilience and disease trajectories remains limited.

**Methods:**

Here, we present the study protocol of the Berlin Research Initiative for Diagnostics, Genetic and Environmental Factors of Schizophrenia (BRIDGE-S), which aims to collect a densely phenotyped genetic cohort of 1,000 schizophrenia cases and 1,000 controls. The study’s main objectives are to build a resource for i) promoting genetic discoveries and ii) genotype–phenotype associations to infer specific disease subtypes, and iii) exploring gene-environment interactions using polyrisk models. All subjects provide a biological sample for genotyping and complete a core questionnaire capturing a variety of environmental exposures, demographic, psychological and health data. Approximately 50% of individuals in the sample will further undergo a comprehensive clinical and neurocognitive assessment.

**Discussion:**

With BRIDGE-S we created a valuable database to study genomic and environmental contributions to schizophrenia risk, onset, and outcomes. Results of the BRIDGE-S study could yield insights into the etiological mechanisms of schizophrenia that could ultimately inform risk prediction, and early intervention and treatment strategies.

**Supplementary Information:**

The online version contains supplementary material available at 10.1186/s12888-022-04447-4.

## Background

Schizophrenia is a clinically heterogeneous psychiatric disorder with a substantial underlying genetic component. Genome-wide association studies identified over 200 common variants, each conferring a small risk [1] and few but high-impact rare variants [2, 3] reflecting a complex molecular architecture and a high degree of polygenicity. The fraction of variance in disease risk attributable to common genetic variation, known as SNP-based heritability, is estimated at 24%, well below the benchmark of 80% derived from a collection of twin and family studies [4, 5]. This discrepancy might partially be explained by the insufficient statistical power to detect all genetic signals and the inflation of twin-based heritability measures that do not account for shared environment within families [6]. Indeed, epidemiological findings further point to multiple environmental exposures with moderate effect sizes that affect schizophrenia onset. These include minority status, urban upbringing, cannabis use, perinatal complications, and childhood adversity, which likely depend on an individual’s genetic vulnerability [7–9].

Despite progress in establishing environmental and genetic factors, our understanding of their individual and combined effects on the disease risk, onset, and outcome is still limited [10]. Theoretical considerations on etiological models postulating an interaction of biological predisposition and external stressors that increase susceptibility to schizophrenia can be traced back to the 1950s [11]. Empirical investigations of gene-environment interactions have proven to be challenging primarily due to a lack of data sources, poor reproducibility of earlier candidate gene studies, and the use of proxy measures like family history [12, 13].

As the focus of research has shifted from single-gene-environmental analyses to polygenic models [14], new approaches and methods are increasingly incorporated, such as Polygenic Risk Scores (PRS) that aggregate the weighted effects of many genetic variants to obtain an overall measure of propensity towards a given trait. A landmark study published in 2019 by EU-GEI investigators provides direct evidence for the additive effects of gene-environment interaction for SCZ-PRS with cannabis use and exposure to childhood adversities [15]. Efforts to substantiate an interplay between polygenic risk and obstetric complications have thus yielded inconsistent results [16, 17]. Similar to PRS, the concept of composite scores of environmental exposures evolved as a tool to capture the cumulative effects of the exposome [18, 19]. Recent findings indicate a higher burden of environmental exposures in first episode patients [20] and individuals with schizophrenia [21]. This might hold particularly true for affected individuals with an earlier onset [22]. Moreover, there is evidence that the effects of environmental exposures and genetic liability on the outcome are not independent [20, 23]. Adding further to the complexity, internal and external protective factors like social support mitigate the influence of genetic vulnerability and adversities to promote positive mental health outcomes and recovery [24]. Determinants contributing to resilience could potentially elucidate differences in disease courses and severity; however, the biological mechanisms underlying such associations remain largely elusive.

Although international consortia like the Psychiatric Genomics Consortium (PGC) have collated many genetic samples, they often lack dense, homogeneous phenotype data needed to assess the functional impact of genetic variation alone and in conjunction with environmental factors. To our knowledge, only a few studies exist that allow for such investigations [25–27], and further research and replication of previous findings are urgently warranted. With the introduction of the BRIDGE-S, we attempt to assemble a large, well-characterized cohort of affected and unaffected individuals to study relationships and interactions between genetics, environment, and phenotypic variance. The purpose of this protocol is to outline scientific objectives and study procedures which will also serve as a foundation for future collaboration.

## Methods

### Aim and objectives

Building on prior research, the primary goal of BRIDGE-S is to recruit a large sample of schizophrenia patients (*N* = 1,000) and unaffected individuals (*N* = 1,000) with comprehensive phenotypic and environmental information alongside genomic data. We aim to build a resource to i) facilitate genetic discoveries ii) study genotype–phenotype relationships within schizophrenia as well as unaffected individuals iii) explore joint and independent effects of environmental and genetic factors that confer risk and resilience on schizophrenia onset and outcomes iv) enable prospective recall studies informed by genotypic and environmental constellations.

### Study design

**Fig. 1:**
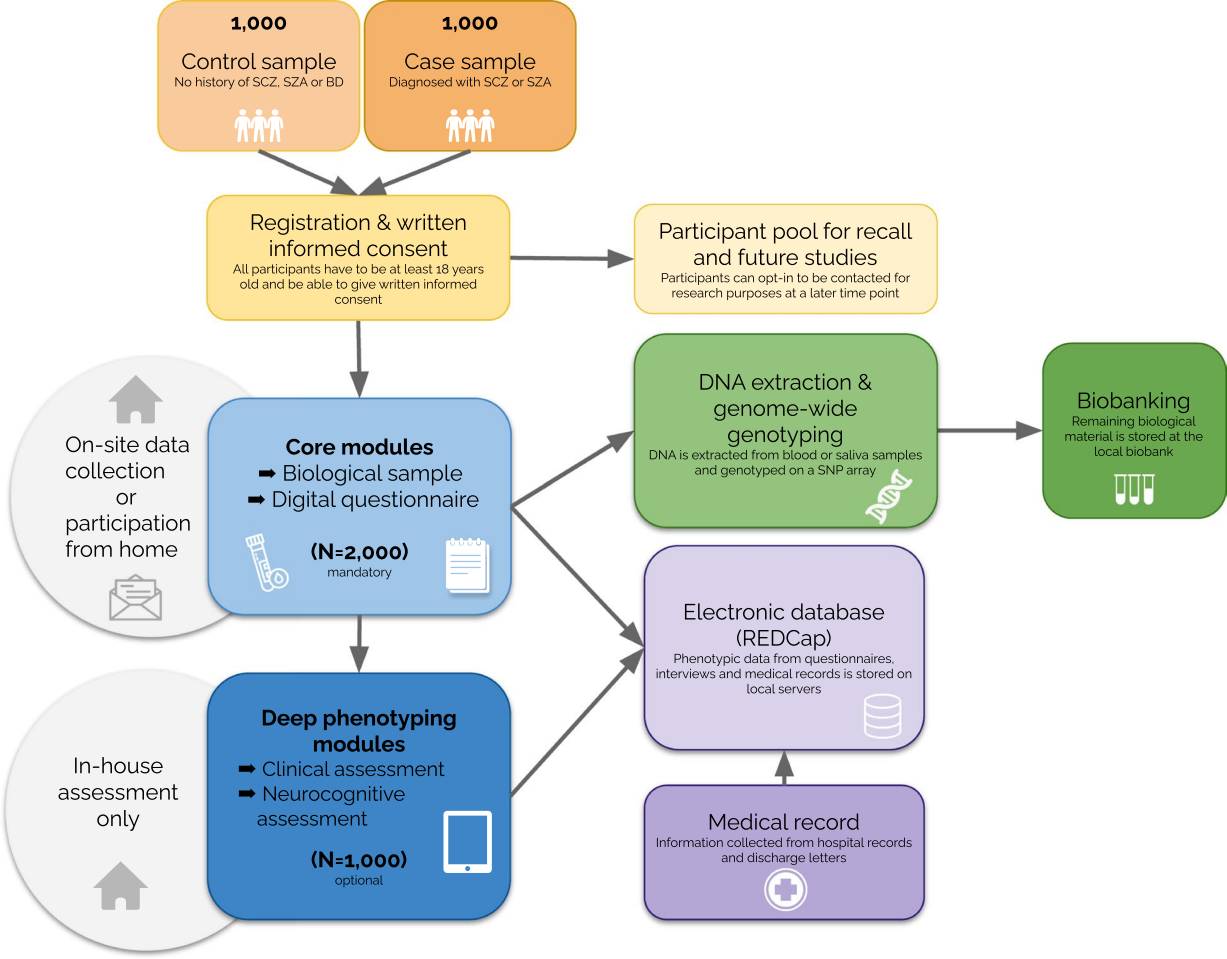
Study design and workflow. Abbreviations: SCZ—Schizophrenia, SZA—Schizoaffective Disorder, BD—Bipolar Disorder

The BRIDGE-S is an ongoing case–control study with a strong focus on accessibility and feasibility. Thus, we established a modular multistage data collection strategy encompassing core and optional modules (see section *Modular Phenotyping*), while participants may choose between in-house assessment and remote participation (see section *In-house assessment and remote participation*). Procedures for cases and controls are very closely aligned, as illustrated in the BRIDGE-S’s study design and workflow in Fig. 1.

During a pilot phase conducted between July 2018 and December 2019, participants were enrolled at two sites to test the feasibility of recruitment, evaluate intended data collection procedures in different settings, and identify solutions to potential issues. We established Standard Operating Procedures (SOPs) to ensure high comparability of collected data across and within study sites.

### Inclusion criteria

All participants must a) be at least 18 years old, b) have sufficient German language skills required to understand the scope of the study and to complete the questionnaires, and c) provide written informed consent. Individuals are allocated to the case sample if they ever met diagnostic criteria for Schizophrenia (ICD-10: F20) or Schizoaffective Disorder (ICD-10: F25) at any point during their life. Formal diagnosis is ascertained upon referral by clinicians via access to medical records or hospital discharge letters. Control subjects are eligible to participate if they have never been diagnosed with Schizophrenia, Schizoaffective-, or Bipolar Disorder (ICD-10: F31); the latter due to its high genetic correlation with Schizophrenia [28]. Non-European ancestry is not an exclusion criterion to promote the recruitment of individuals from all populations including those underrepresented in genetic studies.

### Recruitment

#### Case sample

Study proceedings, especially recruitment strategies, were developed to engage patients from different social backgrounds and with various outcomes. Research team members recruit patients from three core study sites at Charité Universitätsmedizin—Campus Mitte, Charité Universitätsmedizin—Campus Benjamin Franklin, and the Alexianer St. Hedwig Hospital in Berlin, Germany. Several other outpatient clinics and medical practices in the Berlin metropolitan area refer patients by handing out study material. Schizophrenia cases from the broader population are recruited via online advertising on search engines and our institution’s website. Additionally, we launched an advertising campaign in public transportation in November 2021. Patients who participated in an earlier study (the “Berlin Psychosis Study”—BePS) and consented to be contacted for future studies were invited to participate in the present study. These patients were originally recruited through a network of collaborating hospitals in Berlin. BePS focused on the genetic underpinnings of Schizophrenia, most patients already provided a saliva sample for genetic analyses (see section Genetic data & genotyping).

#### Control sample

Healthy controls are recruited from local universities and the broad population via media outlets, including television, newspapers, and radio broadcasts, along with ads on social media platforms and search engines.

### Data collection

#### Registration, informed consent and contact data

Participants are either recruited directly via the clinic or sign up for the study via E-Mail, telephone, or through the registration form on the study website. Upon inclusion, all subjects are asked whether they permit to be contacted for follow-up studies. If participants agree to be informed about future opportunities to take part in research studies, contact information is recorded and stored separately from any biological and health data. Identifying data is processed in compliance with existing data protection laws and access to personal data is restricted to designated staff members.

#### Modular phenotyping

As part of the core modules, all participants provide a biosample for genetic analyses and complete a comprehensive questionnaire. It takes between 45 and 75 min to complete the mandatory core modules. The questionnaire was carefully composed to assess a range of self-report measures required for large-scale investigations while keeping the overall length short. This approach also enables patients with a higher disease burden to engage in the study.

The deep phenotyping module was designed to facilitate secondary analyses on genetic contributions to specific symptom dimensions and clusters. Subjects are assessed regarding symptom severity, cognitive- and overall level of functioning. This module includes a clinical interview for patients, additional questionnaires, and a neurocognitive battery for both cases and control subjects. The optional deep phenotyping modules take ~ 100 min for control subjects and 150–180 min for cases to complete. Based on previous experience we anticipate that ~ 50% of enrolled subjects will complete the deep phenotyping modules.

#### Digital phenotyping & database

Phenotypic data is collected and managed using Research Electronic Data Capture [29, 30] hosted at servers of Charité – Universitätsmedizin Berlin. REDCap is a secure, self-hosted, and web-based software platform developed to support data capture for research studies and clinical trials. In most instances, subjects directly enter questionnaire data into REDCap using the online survey mode. Alternatively, data from printed questionnaires are entered manually into the REDCap database, each data entry is then carefully double-checked by another research team member. If available, interview-based ratings are recorded in corresponding REDCap data entry forms. Real-time data validation and quality rules were defined to ensure that data is entered accurately and as completely as possible.

#### In-house assessment and remote participation

Core modules may be completed from home to lower the barrier to participation for patients that would otherwise not opt for or be able to join an on-site assessment. After registration and contact with the research team, detailed study information, a consent form, and a DNA saliva kit are sent via mail. Patients may choose to fill out the questionnaire as a paper and pencil version from home or as an online survey, in which the eCRF can be accessed through a user-friendly interface. Additional instructions are displayed on the questionnaire landing page and the cover letter that is forwarded together with the saliva kit and consent form. The research team offers support and assists during at-home participation whenever needed.

Participation in the deep phenotyping modules requires an in-house assessment at one of the study sites. For that purpose, participants are reimbursed for any travel expenses in addition to the monetary compensation they receive for their study participation. Participants are eligible to take part in the optional phenotyping modules once the core modules are completed.

#### Questionnaire

The core questionnaire is composed of two parts. The first part may be conducted as an interview and contains questions about socio-demographics, complications during pregnancy and childbirth, parental age at birth, birth month, migration, urbanicity, drug use, basic clinical data, physical health, including traumatic brain injury, as well as family history of certain medical conditions. Basic clinical data that is collected from patients and by chart review contains hospitalizations, age of onset, duration of illness, principal diagnosis, and psychiatric comorbidity, current and past treatments, particularly medication, treatment with clozapine, and electroconvulsive therapy. The second questionnaire part encompasses a selection of self-report instruments. These capture traumatic or adverse events during childhood [31] and across the lifespan [32], resilience [33, 34], social support [35], suicidality [36] subjective well-being [37] and current anxiety and depressive symptoms [38]. Control subjects complete a series of additional questionnaires assessing psychotic-like experiences [39, 40] and schizotypal personality traits in the general population [41] as well as previous or current manic episodes [42].

Besides their appropriateness to assess previously reported risk- and resilience factors and important outcomes in schizophrenia, instruments for phenotyping were selected based on the following aspects: i) length and duration of administration ii) validity and reliability in German-speaking samples iii) applicability across cultures and countries iv) validity and appropriateness for both clinical and population-based samples. A complete list of all instruments administered to the case and control study arms is presented in Table 1.

**Table 1:** Overview of collected data and phenotyping instruments

	**Instrument(s)**	**Data source**	**Cases**	**Controls**
**Core questionnaire**				
Socio-demographics		SR/I	x	x
Complications during pregnancy and birth		SR/I/MR	x	x
Seasonality		SR/I	x	x
Urbanicity		SR/I	x	x
Migration		SR/I	x	x
History of drug use		SR/I/MR	x	x
History of mental illness		SR/I		x
Basic clinical data		SR/I/MR	x	
Health data		SR/I/MR	x	x
Childhood adversity	CTQ	SR	x	x
Lifetime traumatic events	ETI^a,b^	SR	x	x
Psychotic experiences in the population	LSHS-R, PDI-k21, PCL-5^a^	SR		x
Schizotypy	SPQ-BR	SR		x
Subclinical (hypo)mania	MDQ	SR		x
Suicidality	SBQ-R	SR	x	x
Resilience	RS-13, BRS	SR	x	x
Social support	FSozU-k14	SR	x	x
Anxiety & Depression	PHQ-4	SR	x	x
Subjective wellbeing	PWI-A	SR	x	x
**Neurocognitive assessment**				
Sensorimotor function, comprehension	MOT	TB	x	x
Processing & psychomotor speed	RTI	TB	x	x
Working Memory & strategy	SWM	TB	x	x
Verbal memory and new learning	VRM	TB	x	x
Visual episodic memory	PAL	TB	x	x
Planning	OTS	TB	x	x
Sustained Attention	RVP	TB	x	x
Emotion Recognition	ERT	TB	x	x
Multitasking	MTT	TB	x	x
Handedness	EHI-SF	SR	x	x
**Clinical assessment**				
Symptom profile	PANSS	CR	x	
Symptom Severity	CGI-S	CR	x	
Psychopathology	SCL-90-R	SR	x	x
Global functioning	GAF	CR	x	
Disability and functional outcomes	WHODAS 2.0	SR/I	x	x

^a^Original instrument was adapted, ^b^Similar to the Life Events Checklist for DSM-5 (LEC-5)

*SR* Self-reported, *I* Interview, *CR* Clinician rated, *MR* Medical records, *TB* Tablet based

#### Genetic data & genotyping

All study subjects provide a biological sample for genome-wide genotyping. Most subjects donate a 1.0 ml saliva sample using OraGene-510 or OraGene-610 DNA-Self-Collection Kits (Genotek, Ottawa, Ontario, Canada), which is an easy, safe, and user-friendly collection system that can also be used for self-administration at home. Alternatively, a blood sample (EDTA whole-blood, commercially available brands) is collected during routine care laboratory blood sampling. Saliva and blood samples are stored on-site until further processing.

DNA is extracted from saliva and blood samples following established standard protocols in Berlin, Germany. DNA stock solutions are transferred to the central Charité biobank (ZeBanC) for long-term storage at -60 degrees. Normalized DNA aliquots are sent to the ERASMUS Medical Center’s Human Genotyping Facility (HuGe-F) in the Netherlands for genotyping. All samples are assayed on the Illumina Infinium Global Screening Array (GSA) MD BeadChip (Illumina, San Diego, CA), which covers > 650,000 genetic variants. Genome-wide SNP data is processed on an High Performance Computing cluster.

#### Neuropsychological assessment

Participants undergo a neurocognitive assessment to measure performance in different domains. The battery encompasses tests measuring memory capacity, executive functioning, decision making, social cognition, attention, and psychomotor speed. To assure consistency and standardization across multiple sites, we adopted a fully computerized neurocognitive battery using the Cambridge Neuropsychological Test Automated Battery (CANTAB; [43]) system.

The CANTAB schizophrenia battery is composed of eight tasks covering key domains recommended by the MATRICS initiative (Marder, 2006): Reaction Time (RTI), Rapid Visual Information Processing (RVP), Paired Associates Learning (PAL), Verbal Recognition Memory (VRM), Spatial Working Memory (SWM), Multitasking Test (MTT), One Touch Stockings of Cambridge (OTS), and Emotion Recognition Task (ERT). The battery was preceded by a short Motor Screening Task (MOT) to familiarize participants with the setting & usage and screen for potential motor and comprehension issues. An overview of the test battery and domains assessed is shown in Table 1. Respective test versions and sequences applied in the current study can be found in Supplementary Table 1.

Instructions are presented via voice-over in German for each test. Subjects interact with a touchscreen system on a 10.5–11 inch display (Apple iPad, iOS version 12.1 or later). The entire battery takes ~ 75 min to complete. Key outcome measures are automatically recorded and stored on secure servers hosted by Cambridge Cognition. In addition to CANTAB, the short version of the Edinburgh Handedness Inventory (EHI-SF) [44] is administered as part of the module.

#### Clinical assessment

Assessment of clinical symptoms differs between cases and controls (see Table 1). The Positive and Negative Syndrome Scale (PANSS) [45] is used as the primary measure to examine the severity of specific symptoms associated with schizophrenia during the past seven days. The scale is rated by trained clinical staff based on a semi-structured interview (SCI-PANSS) that typically takes between 45–90 min. Wherever possible, information from family members, caregivers, or hospital staff is gathered to rate items requiring a third-person perspective adequately. At least 10% of all PANSS interviews are rated independently by two members of the research team to ensure overall consistency between raters and calculate the inter-rater reliability. In addition to PANSS scores, the Clinical Global Impression (CGI-S) [46] and global assessment of functioning (GAF) [47] were used to measure global symptom burden and level of functioning, respectively. Finally, both cases and controls complete the WHO disability schedule (WHODAS 2.0) [48], and the Symptom Checklist 90 Revised (SCL-90-R) [49, 50].

### Statistical analysis plan

#### Facilitating genetic discoveries (Aim 1)

Statistical analysis of genome-wide SNP data will be conducted using standard software like PLINK [51] and RICOPILI [52], a pipeline that allows for standardized and efficient common variant analyses at all steps: quality control (QC), relatedness testing & principal component analysis (PCA), genotype imputation, and association analysis. Stringent QC filters incorporated in RICOPILI will be applied to obtain high-quality genetic data for analyses. Imputed genotype data will be meta-analyzed with other samples aggregated by PGC investigators to i) identify genomic loci associated with schizophrenia risk ii) to dissect disorder-specific and shared SNP associations across multiple or pairs of psychiatric disorders and other relevant phenotypes in cross-trait approaches and iii) to uncover genetic variation underlying specific dimensional phenotypes within and beyond diagnostic categories, e.g., exploring symptom profiles, treatment outcomes. A range of post-GWAS analyses will be performed on summary-level data to increase interpretability, e.g., gene-set analysis and genetic correlations to quantify the molecular overlap between traits.

#### Genotype–phenotype analyses (Aim 2)

Complementing Aim 1, we will calculate PRS that index liability to various disorders and traits in our target sample, following a leave-one-out approach whenever appropriate to avoid overlap with discovery samples. Polygenic associations will be tested for multiple subjective and objective outcome measures. We will particularly focus on the level of functioning, cognitive markers, and clinical dimensions in patients. Complimentarily, we will investigate schizotypy and psychotic-like experiences like hallucinations and delusions and their relationship with genetic risk in the control sample.

#### Gene-environment analyses (Aim 3)

We further aim to explore genomic and environmental influences on schizophrenia risk, the occurrence of psychotic symptoms, and other secondary phenotypes by examining main and additive interaction effects between i) polygenic risk and specific exposures as well as environmental scores (ES) that combine individual effects of exposures ii) pathway-based PRS, that aggregate genetic risk across distinct biological pathways, ES and single exposures iii) single and multiple determinants that confer risk and resilience in an integrative model. Besides gene-by-environment interaction, we will also assess gene-environment correlations. Furthermore, we will incorporate state-of-the-art methods to compute ES [53] and to estimate the causal effects of environmental factors by leveraging genetic risk variants, for instance, via Mendelian Randomization [54].

#### Sample size and power calculation

Sample size for the main effects was calculated according to Cohen [55]. We expect small to modest effect sizes in analyses with dichotomous and continuous outcomes and PRS and ES environmental exposures as predictors. By including *N* = 2,000 (1,000 cases and 1,000 controls) into the core module, we will be able to detect potentially very small main effects (f^2^ ≥ 0.005, OR ≥ 1.29 at *p* ≤ 0.05) with sufficient power of ≥ 80% as visualized in Supplementary Figure S1. Further, the planned sample size is sufficient to test case-only (*N* = 1000) hypotheses and conduct analyses limited to the deep phenotyping module (*N* = 500/500) while still detecting small effects of (f^2^ ≥ 0.01, OR ≥ 1.44, *p* ≤ 0.05) with a power of ≥ 80%. According to VanderWeele’s method [56], the proposed sample size is suitable to detect a positive additive interaction in the case–control sample, assuming a rare outcome (main effects OR = 1.3; IOR = 1.5; *p* ≤ 0.05; power = 80%).

## Discussion

Current evidence highlights the complex and multifactorial etiology of schizophrenia [13]. In addition to expanding our genetic knowledge of schizophrenia and related disorders, our study may provide valuable insights into molecular pathways that underlie variability in psychopathology, disease course, and other important outcomes. Importantly, our database enables us to test etiological hypotheses in the context of schizophrenia and emerging subclinical psychotic symptoms involving environmental and genetic factors. A better understanding of etiopathogenic processes is likely to inform precision-medicine approaches, particularly personalized prevention, and therapy strategies. While patient-tailored prediction models integrating biological, environmental, and lifestyle factors are common practice in the diagnosis and management of other disorders, for example, cardiovascular disease [57], such measures still have to be established within psychiatry.

Within BRIDGE-S, we designed a flexible study framework to efficiently recruit a large number of patients while collecting a broad selection of phenotypes by implementing a modular workflow, remote participation, and digital tools. However, compromises between maximizing sample size and depth of phenotyping were made on several levels, including the omission of structured diagnostic interviews in favor of shorter self-reported instruments and chart reviews for case ascertainment. By employing a condensed questionnaire for the initial assessment, we also sought to improve study enrollment and counteract a putative selection bias.

At present, this study does not generate longitudinal data, but follow-up visits are possible through the recall of selected participants. This represents an excellent opportunity to acquire additional biological samples for further omics analyses or information to map disease trajectories, but also to address more specific research questions by targeting individuals with distinct genetic or environmental configurations. Such approaches can be advantageous to efficiently and causally interrogate biological mechanisms behind genetic associations and trial personalized treatment concepts [58].

## Supplementary Information

**Additional file 1: Table S1.** CANTAB battery and test variants (completed in the exact same order). **Figure S1.** A priori sample size calculation based on expected small effect sizes.

## Data Availability

To promote reproducibility and collaborations, additional study materials and resources such as variable lists, data codebook, metadata, and analysis scripts will be shared on a public GitHub repository after completion of the study and initial dissemination: https://github.com/Ripkelab. Summary-level genetic data will be shared publicly via established repositories such as GWAS Catalog, meta-analyses summary statistics will be available for download via the Psychiatric Genomics Consortium (PGC). Genotype data at the individual level can be obtained upon reasonable request either through the authors or the Data Access Committee (DAC) of PGC. Please reach out to the corresponding author and principal investigator of the study if you are interested in collaborating or working with primary data from BRIDGE-S.

## Abbreviations

BD: Bipolar Disorder
BRIDGE-S: Berlin Research Initiative for Diagnostics, Genetics and Environmental Factors in Schizophrenia
BRS: Brief Resilience Scale
CANTAB: Cambridge Neuropsychological Test Automated Battery
CGI-S: Clinical Global Impression Scale (severity)
GAF: Global Assessment of Functioning
GSA: Global Screening Array
CTQ: Childhood Trauma Questionnaire
GWAS: Genome-wide Association Study
ERT: Emotion Recognition Task
ETI: Essener Trauma Inventar
ES: Environmental scores
EU-GEI: EUropean Network of National Schizophrenia Networks Studying Gene–Environment Interactions
FSozU-k14: Fragebogen zur Sozialen Unterstützung 14-item version (Engl.: Perceived Social Support Questionnaire 14-item version)
ICD: International Statistical Classification of Diseases and Related Health Problems
IOR: Odds ratio multiplicative interaction
LSHS: Launay-Slade Hallucination Scale
MATRICS: Measurement and Treatment Research to Improve Cognition in Schizophrenia (initiative)
MDQ: Mood Disorder Questionnaire
MOT: Motor Screening Task
MTT: Multitasking Test
OR: Odds Ratio
OTS: One Touch Stockings of Cambridge
PAL: Paired Associates Learning
PANSS: Positive and Negative Syndrome Scale
PCA: Principal Component Analysis
PCL-5: Paranoia Checklist 5
PDI-k21: Peters et al. Delusion Inventory 21-item version
PHQ-4: Patient Health Questionnaire 4-item version
PRS: Polygenic Risk Scores
PGC: Psychiatric Genomics Consortium
PWI-A: Personal-Wellbeing-Index for Adults
QC: Quality Control
RICOPILI: Rapid Imputation for COnsortias PIpeLIne for GWAS
RS-13: Resilience Scale 13-item version
RTI: Reaction Time
RVP: Rapid Visual Information Processing
SBQ-R: Suicidal Behavior Questionnaire
SCL-90-R: Symptom Checklist 90 item revised
SCZ: Schizophrenia
SNP: Single Nucleotide Polymorphism
SOP: Standard Operation Procedure
SPQ-BR: Schizotypal Personality Questionnaire Brief Revised
SWM: Spatial Working Memory
SZA: Schizoaffective Disorder
VRM: Verbal Recognition Memory
WHODAS 2.0: WHO Disability Assessment Schedule 2.0
