# Supplementary material for: Study protocol of the Berlin Research Initiative for Diagnostics, Genetics and Environmental Factors in Schizophrenia (BRIDGE-S)
Source: BMC Psychiatry. 2023 Jan 12;23:31. doi: 10.1186/s12888-022-04447-4 (PMC9835268; doi:10.1186/s12888-022-04447-4)

Supplementary Material

Table S1. CANTAB battery and test variants (completed in the exact same order)

**Test Code**	**Variant**	**Cognitive process**
MOT	Voice	Sensorimotor function / comprehension
RTI	Five-Choice	Processing and psychomotor speed
SWM	Recommended standard 2.0	Working memory and strategy
VRM	Recommended Standard (immediate)	Verbal memory and new learning
PAL	Recommended Standard Extended	Visual episodic memory
OTS	Standard	Planning
RVP	3 Targets	Sustained Attention
ERT	short	Emotion Recognition
VRM (recommended Standard delayed)		
MTT	Standard	Multitasking

Figure S1. A prioiri sample size calculation based on expected small effect sizes

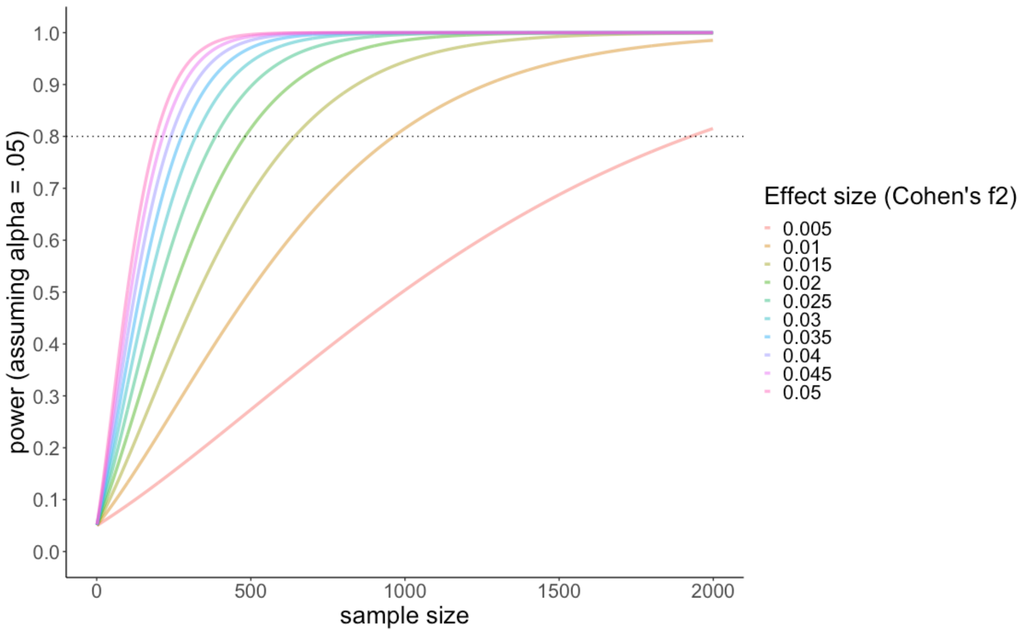

Supplement: Supplementary file 1 — Additional file 1: Table S1. CANTAB battery and test variants (completed in the exact same order). Figure S1. A priori sample size calculation based on expected small effect sizes. [file 12888_2022_4447_MOESM1_ESM.pdf]
